# Genetic modification of obesity-associated disability progression in multiple sclerosis

**DOI:** 10.1007/s00415-026-14091-7

**Published:** 2026-09-08

**Authors:** Jie Guo, Jing Wu, Tomas Olsson, Lars Alfredsson, Anna Karin Hedström

**Affiliations:** 1 Department of Clinical Neuroscience, Karolinska Institutet, CMM, L8, 171 76 Stockholm, Sweden; 2 Department of Nutrition and Health, China Agricultural University, Beijing, China; 3 Institute of Environmental Medicine, Karolinska Institutet, Stockholm, Sweden; 4 Centre for Occupational and Environmental Medicine, Region Stockholm, Stockholm, Sweden

**Keywords:** Multiple sclerosis, Human leukocyte antigen, Genetic modification, Body mass index, Disability progression, Expanded disability status scale

## Abstract

**Background:**

Obesity has been associated with adverse outcomes in multiple sclerosis (MS), but whether its influence on disability progression differs according to immunogenetic background remains unclear. We investigated whether associations between obesity at diagnosis and subsequent disability progression vary by HLA-A*02:01 status.

**Methods:**

Using data from two Swedish population-based case–control studies, we included 2117 individuals with MS, disease duration ≤5 years at study inclusion, and available HLA genotyping. Obesity was defined as body mass index (BMI) >30 kg/m^2^. Participants were followed through the Swedish MS registry for confirmed disability worsening (CDW), time to Expanded Disability Status Scale (EDSS) 3 and 4, and longitudinal Expanded Disability Status Scale (EDSS) trajectories. Hazard ratios (HRs) were estimated using adjusted Cox regression models, and mixed-effects models were used to evaluate disability trajectories over time.

**Results:**

Among individuals lacking HLA-A*02:01, obesity was associated with an increased risk of CDW (HR 1.49, 95% CI 1.12–1.82), EDSS 3 (HR 1.90, 95% CI 1.30–2.79), and EDSS 4 (HR 2.30, 95% CI 1.33–3.73). No corresponding associations were observed among HLA-A02:01-positive individuals. Analyses treating BMI as a continuous variable yielded similar findings. Additive interaction analyses further supported a joint effect of obesity and absence of HLA-A*02:01 on disability progression. Longitudinal models demonstrated the highest predicted EDSS trajectories among obese HLA-A*02:01-negative individuals.

**Conclusions:**

Obesity at diagnosis was associated with worse disability outcomes primarily among individuals lacking HLA-A*02:01, suggesting that HLA class I-related immune pathways may modify the influence of excess adiposity on MS disease progression.

**Supplementary Information:**

The online version contains supplementary material available at https://doi.org/10.1007/s00415-026-14091-7.

## Background

Multiple sclerosis (MS) is a chronic inflammatory and neurodegenerative disease of the central nervous system and a leading cause of non-traumatic neurological disability in young adults. Although the etiology of MS reflects complex interactions between genetic susceptibility and environmental exposures [1–3] far less is known about how such exposures influence disability progression.

Among modifiable lifestyle-related factors, obesity has emerged as a potentially important determinant of disease course in MS [1–3]. In addition to its established role in MS susceptibility, obesity has been associated with faster disability accumulation and worse clinical outcomes [4–6]. Adipose tissue functions as an active immunometabolic organ that promotes chronic low-grade inflammation, altered adipokine signalling, and immune dysregulation, mechanisms that may contribute to neuroinflammatory processes and disability progression [7–9].

Genetic factors are thought to play a relatively modest role in determining long-term disability progression in MS. Genome-wide studies have identified few severity-associated loci [10], and polygenic risk scores derived from susceptibility markers show limited predictive value for disability trajectories [11]. Nonetheless, specific genetic backgrounds may still influence how individuals respond to environmental exposures even in the absence of strong marginal genetic effects. Recent findings that smoking-related disability progression is largely confined to individuals lacking HLA-A*02:01 [12] raises the possibility that genetically defined immune pathways influence responses to environmental factors after disease onset.

Whether the adverse effects of obesity on disability progression differ according to the HLA genotype is unclear. In the present study, we followed 2117 newly diagnosed MS cases to investigate whether the association between obesity at diagnosis and subsequent disability progression differs according to the HLA-A*02:01 status.

## Methods

Our study comprised patients from the Swedish population-based case–control studies Epidemiologic Investigation of Multiple Sclerosis (EIMS) [13] and Genes and Environment in Multiple Sclerosis (GEMS) [13]. Incident cases were recruited to EIMS from 40 hospital-based neurology units across Sweden, including all university hospitals, between April 2005 and December 2015 (*n*=2424). Eligibility criteria for cases were age 16–70 years, residence in Sweden, and a neurologist-confirmed diagnosis of MS according to the McDonald criteria, as applicable at the time of diagnosis [14, 15].

GEMS recruited 6148 prevalent cases of MS who were not participating in EIMS through the Swedish MS registry between November 2009 and November 2011. The participation rate was 93% for EIMS and 82% for GEMS. To ensure that body mass index (BMI) reflected body weight relatively close to the time of diagnosis and to reduce the potential influence of long-standing disease on body weight, analyses were restricted to participants with a disease duration of five years or less at study inclusion. This criterion was fulfilled by 2247 EIMS participants and 741 GEMS participants. Among these, HLA was available for 2117 participants (1527 from EIMS and 590 from GEMS). A flowchart describing participant selection and the derivation of the analytical sample is presented in eFigure 1.

Standard Protocol Approvals, Registrations, and Patient Consents.

The studies were approved by the Regional Ethical Review Board at Karolinska Institute and have been performed in accordance with the ethical standards laid down in the 1964 Declaration of Helsinki and its later amendments (reference number: 04–252/1–4 and 2008/1617–31/2). Participants provided written informed consent.

### Definition of exposures and covariates

Information on environmental exposures and lifestyle habits was collected using an extensive questionnaire. The primary exposure was obesity at study inclusion. BMI was calculated from self-reported height and weight and categorized as obese (BMI>30 kg/m^2^) or non-obese (BMI≤30 kg/m^2^).

Alcohol consumption was categorized as no consumption, low, moderate, or high consumption according to cutoffs used by Statistics Sweden [16]. Sun exposure habits were assessed using two four-level items. Scores were summed to a total index ranging from 2 to 8 and dichotomized into low (score 2) and high (score >2) levels. Participants reported their typical physical activity level during the previous year by selecting one of four pre-defined categories ranging from predominantly sedentary behavior to regular, sweat-inducing exercise at least 3 times per week. Physical activity was dichotomized into low (original categories 1–2) and high (categories 3–4).

HLA alleles were identified at four-digit resolution. Genotyping was conducted using the MS replication chip [17] and HLA allele identification was performed through HLA*IMP:02 imputation [18]. We focused on HLA-A*02:01 because of its established role in MS susceptibility and emerging evidence that it may modify the impact of environmental exposures on disease progression. Participants were categorized according to the presence or absence of each allele.

### Outcome measures

The Swedish MS registry is used nationwide and is integrated into routine clinical documentation [19]. Neurologists prospectively record data on disease activity, neurological functioning, and treatment exposure at each visit, enabling longitudinal evaluation for disability outcomes. For the present analyses, baseline was defined as the date of the first available EDSS assessment. Disability progression was evaluated using confirmed disability worsening (CDW), defined as an increase in EDSS by at least 1 point from baseline, sustained between two follow-up visits separated in time by no less than six months (or by ≥1.5 points if EDSS at baseline was 0, and 0.5 points if the baseline EDSS was ≥ 5.5). Time to reaching confirmed EDSS milestones 3 and 4 was also analyzed, limited to subgroups of patients with a baseline EDSS score of less than 3.

### Statistical analysis

Time-to-event analyses were analyzed using Cox proportional hazards regression to estimate hazard ratios (HRs) with 95% confidence intervals (CIs). Follow-up time was calculated from baseline until the occurrence of the outcome of interest, loss to follow-up, death, or end of study (30 March 2026), whichever came first. Proportional hazard assumptions were evaluated using Schoenfeld residuals, with no evidence of violations. All main models were adjusted for age at baseline, sex, ancestry, disease phenotype, disease duration at baseline, baseline EDSS, smoking status (current vs non-smoking), and category of first disease-modifying therapy (DMT) (none, platform therapy, or high-efficacy DMT).

To assess potential effect modification by HLA-A*02:01, associations between obesity and disability progression were estimated separately among HLA-A*02:01-positive and HLA-A*02:01-negative individuals. BMI was further modeled as a continuous variable to examine exposure–response relationships within each genotype stratum.

Interaction between obesity and HLA-A*02:01 was evaluated by creating four exposure groups, using non-obese individuals with the protective genotype (A*02:01 positive) as the reference category. Kaplan–Meier curves were generated to visualize time to CDW according to obesity and HLA-A*02:01 status. Departure from additivity of effects was assessed using the attributable proportion due to interaction (AP), with 95% CIs derived using the delta method. Additive interaction was chosen because it provides a clinically interpretable measure of whether the combined effect of genotype and exposure exceeds the sum of their individual contributions.

Longitudinal mixed-effects models were utilised as a complementary analysis. The models included the obesity/HLA-A*02:01 groups, time, and group-by-time interaction terms, and were adjusted for the same covariates as in the primary Cox regression analyses. To facilitate interpretation of the longitudinal analyses, between-group differences were estimated from the mixed-effects models at yearly intervals during follow-up.

Since DMT exposure strongly influences disability outcomes, we applied models in which DMT use was treated as a time-variable covariate. Additional models were further adjusted for past infectious mononucleosis (yes, no, unsure), alcohol consumption, sun exposure, and physical activity. All analyses were performed in SAS version 9.4.

## Results

The study population comprised 2117 individuals with newly diagnosed MS. Mean age was 35.5 years at disease onset, 36.7 years at diagnosis, and 38.0 years at study inclusion. Baseline EDSS was recorded 0.2 years (IQR 0.02–0.2) after diagnosis. The median follow-up time was 14.1 years (interquartile range 10.3–17.3). Overall, demographic and clinical characteristics were largely comparable between genotype groups. A higher proportion of A*02:01 positive participants were of Nordic origin. Baseline characteristics, overall and by A*02:01 status, are presented in Table 1.

**Table 1:** Characteristics of overall sample and by HLA-A*02:01 status

		Total	A*02:01 neg	A*02:01 pos	*P* value
N		2117	1226	891	
Age at disease onset (SD)		35.5 (10.9)	35.3 (10.9)	35.9 (10.9)	0.19
Age at diagnosis (SD)		36.7 (11.1)	36.4 (11.2)	37.0 (11.0)	0.19
Age at study inclusion (SD)		38.0 (11.2)	37.7 (11.2)	38.4 (11.2)	0.21
Sex, *n* (%)	Female	1536 (72.6)	889 (72.5)	647 (72.6)	0.96
	Male	581 (27.4)	337 (27.5)	244 (27.4)	
Ancestry, *n* (%)	Nordic	1765 (83.4)	993 (81.0)	772 (86.6)	0.0006
	Non-Nordic	352 (16.6)	233 (19.0)	119 (13.4)	
MS type, *n* (%)	Relapsing-onset	1937 (91.5)	1118 (91.2)	819 (91.9)	0.82
	Progressive-onset	106 (5.0)	63 (5.1)	43 (4.8)	
	Unknown	74 (3.5)	45 (3.7)	29 (3.3)	
DMT, *n* (%)	Never	109 (5.2)	67 (5.5)	42 (4.7)	0.30
	Platform	1748 (82.6)	999 (81.5)	749 (84.1)	
	High-efficacy	260 (12.3)	160 (13.1)	100 (11.2)	
Years from diagnosis to DMT initiation, median (IQR)		0.08 (0.02–0.2)	0.08 (0.02–0.2)	0.08 (0.02–0.2)	0.36
Years from diagnosis to first EDSS, median (IQR)		0.2 (0–1.2)	0.1 (0–1.1)	0.2 (0–1.3)	0.46
Baseline EDSS, mean (SD)		1.9 (1.5)	1.9 (1.6)	1.8 (1.5)	0.46
Baseline EDSS, median (IQR)		1.5 (1.0–2.5)	2.0 (1.0–3.0)	1.5 (1.0–2.5)	
Smoking status, *n* (%)	Never smoker	979 (46.2)	584 (47.6)	395 (44.3)	0.27
	Current smoker	564 (26.6)	323 (26.4)	241 (27.1)	
	Past smoker	574 (27.1)	319 (26.0)	255 (28.6)	
BMI status, *n* (%)	Normal weight	1220 (57.6)	711 (58.0)	509 (57.1)	0.62
	Overweight	590 (27.9)	345 (28.1)	245 (27.5)	
	Obese	307 (14.5)	170 (13.9)	137 (15.4)	
Sun exposure, *n* (%)	Low	384 (18.1)	213 (17.4)	171 (19.2)	0.95
	Moderate-high	1733 (81.9)	1013 (82.6)	720 (80.8)	
Physical activity	Low	1328 (62.7)	756 (61.7)	572 (64.2)	0.23
	Moderate-high	789 (37.3)	470 (38.3)	319 (35.8)	

^1^initial platform therapy followed by high-efficacy DMT. ^1^available for 912 participants diagnosed between 2005 and 2009. *HLA* human leukocyte antigen, *DMT* disease-modifying therapy, *EDSS* Expanded Disability Status Scale, *SD* standard deviation, *BMI* body mass index

Obesity was associated with an increased risk of CDW among individuals lacking HLA-A*02:01 (HR 1.40, 95% CI 1.15–1.72), whereas no corresponding association was observed among HLA-A*02:01-positive individuals (HR 1.14, 95% CI 0.89–1.45) (Table 2).

**Table 2:** Associations between obesity and confirmed disability progression, stratified by HLA-A*02:01

BMI	N	Years (SD)	CDW (%)	HR (95% CI)	HR (95% CI)
HLA-A*02:01 positive					
<30	754	7.6 (6.0)	449 (59.6)	1.0 (reference)	1.0 (reference)
>30	137	6.8 (5.6)	79 (57.7)	1.03 (0.81–1.32)	1.14 (0.89–1.45)
HLA-A*02:01 negative					
<30	1056	7.8 (6.2)	611 (57.9)	1.0 (reference)	1.0 (reference)
>30	170	6.2 (5.6)	115 (67.7)	**1.38 (1.13–1.69)**	**1.40 (1.15–1.72)**

The model was adjusted for age at baseline, sex, ancestry, calendar year of diagnosis, disease phenotype, disease duration, baseline EDSS, smoking status, and DMT exposure. *HLA* human leukocyte antigen, *BMI* body mass index, *EDSS* expanded disability status scale, *HR* hazard ratio, *CI* confidence interval

Bold values indicate statistical significance

In analyses treating BMI as a continuous variable, higher BMI was associated with increased risk of CDW among A*02:01-negative individuals (HR per kg/m^2^ 1.02, 95% CI 1.01–1.03), whereas no association was observed among HLA-A*02:01-positive individuals (HR 1.01, 95% CI 0.99–1.03).

**Fig. 1:**
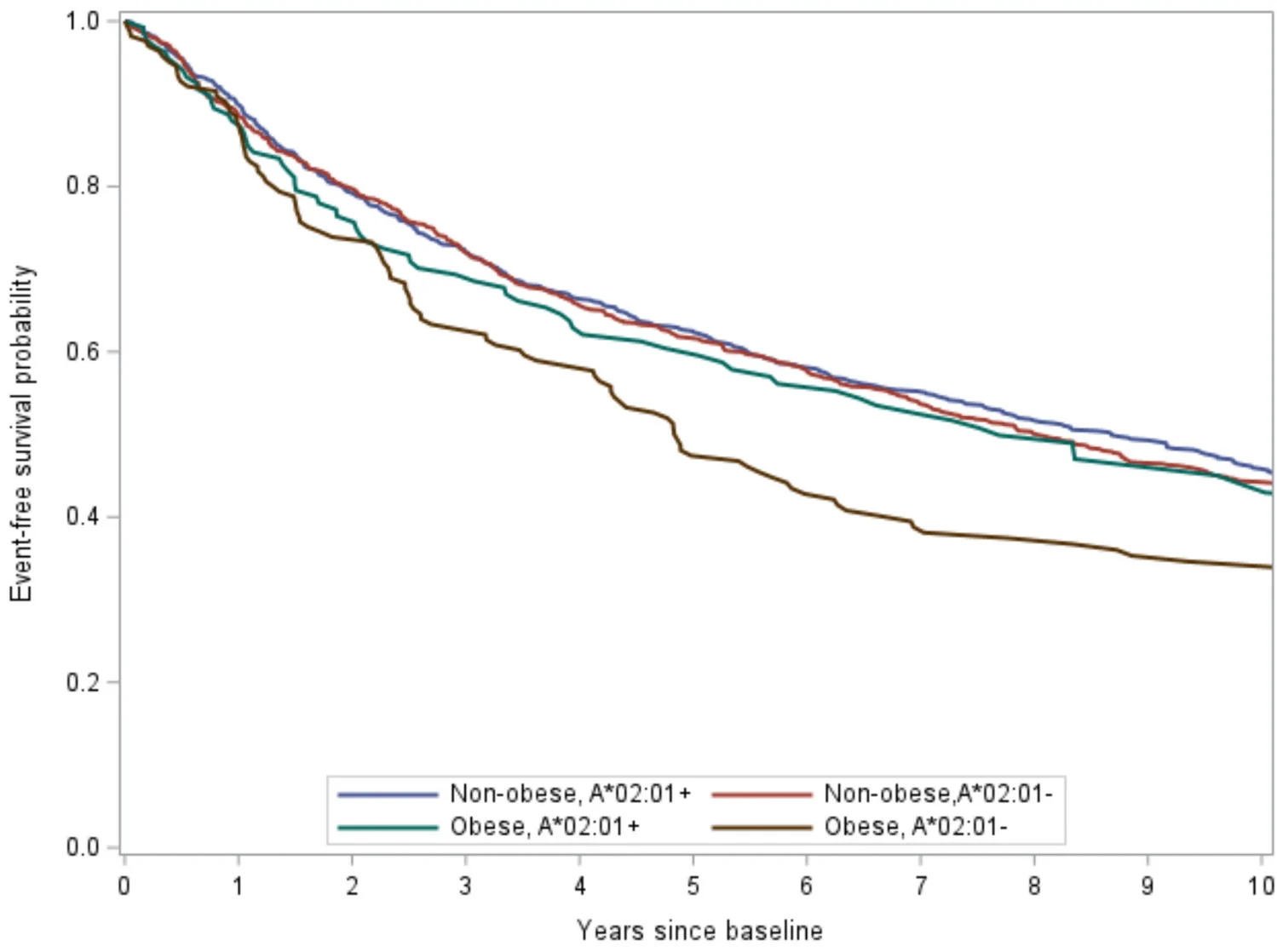
Kaplan–Meier curves for confirmed disability worsening stratified by obesity and HLA-A*02:01 status. *HLA* human leukocyte antigen, *DMT*disease-modifying therapy. The model was adjusted for age at baseline, sex, ancestry, calendar year of diagnosis, disease phenotype, disease duration, baseline EDSS, smoking status, and DMT exposure

Compared with non-obese HLA-A*02:01-positive individuals, HLA-A*02:01-negative individuals with obesity had an increased risk of CDW (HR 1.49, 95% CI 1.12–1.82), EDSS 3 (HR 1.90, 95% CI 1.30–2.79), and EDSS 4 (HR 2.30, 95% CI 1.33–3.73). In contrast, HLA-A*02:01-positive individuals with obesity showed no convincing elevation in risk across any outcome (Table 3Query, Fig. 1). Across outcomes, AP estimates indicated additive interaction between absence of A*02:01 and obesity (AP 0.33, 95% CI 0.03–0.06 for CDW) (eTable 1).

**Table 3:** Associations between obesity and disability progression outcomes by obesity and HLA-A*02:01 status

Obesity	A*02	*N*	Years (SD)	Outcome (%)	HR (95% CI)^1^	HR (95% CI)^2^
First clinical disease worsening (CDW)						
−	+	754	7.6 (6.0)	449 (59.5)	1.0 (reference)	1.0 (reference)
−	−	1056	7.8 (6.2)	611 (57.9)	0.97 (0.87–1.09)	0.99 (0.88–1.12)
+	+	137	6.8 (5.6)	79 (57.7)	1.93 (0.66–1.33)	0.94 (0.63–1.28)
+	−	170	6.2 (5.6)	115 (67.7)	**1.37 (1.04–1.81)**	**1.49 (1.12–1.82)**
EDSS 3						
−	+	583	10.1 (6.2)	226 (38.8)	1.0 (reference)	1.0 (reference)
−	−	784	10.1 (6.3)	315 (40.2)	1.04 (0.89–1.23)	1.00 (0.85–1.17)
+	+	101	8.8 (5.8)	46 (45.5)	1.05 (0.63–1.74)	1.02 (0.62–1.70)
+	−	128	7.8 (5.7)	70 (54.7)	**2.04 (1.40–2.98)**	**1.90 (1.30–2.79)**
EDSS 4						
−	+	583	12.1 (5.9)	107 (18.4)	1.0 (reference)	1.0 (reference)
−	−	784	12.4 (5.8)	153 (19.5)	1.06 (0.84–1.35)	0.99 (0.78–1.26)
+	+	101	11.5 (5.5)	21 (20.8)	1.27 (0.66–2.42)	1.28 (0.79–3.55)
+	−	128	11.0 (5.5)	36 (28.1)	**2.43 (1.46–4.05)**	**2.30 (1.33–3.73)**

^1^adjusted for sex and age at baseline; ^2^adjusted for age at baseline, sex, ancestry, calendar year of diagnosis, disease phenotype, disease duration, baseline EDSS, smoking status, and DMT exposure. *HR* hazard ratio, *CI* confidence interval, *CDW* confirmed disability worsening, *EDSS* expanded disability status scale, *HLA* human leukocyte antigen, *SD* standard deviation

Bold values indicate statistical significance

**Fig. 2:**
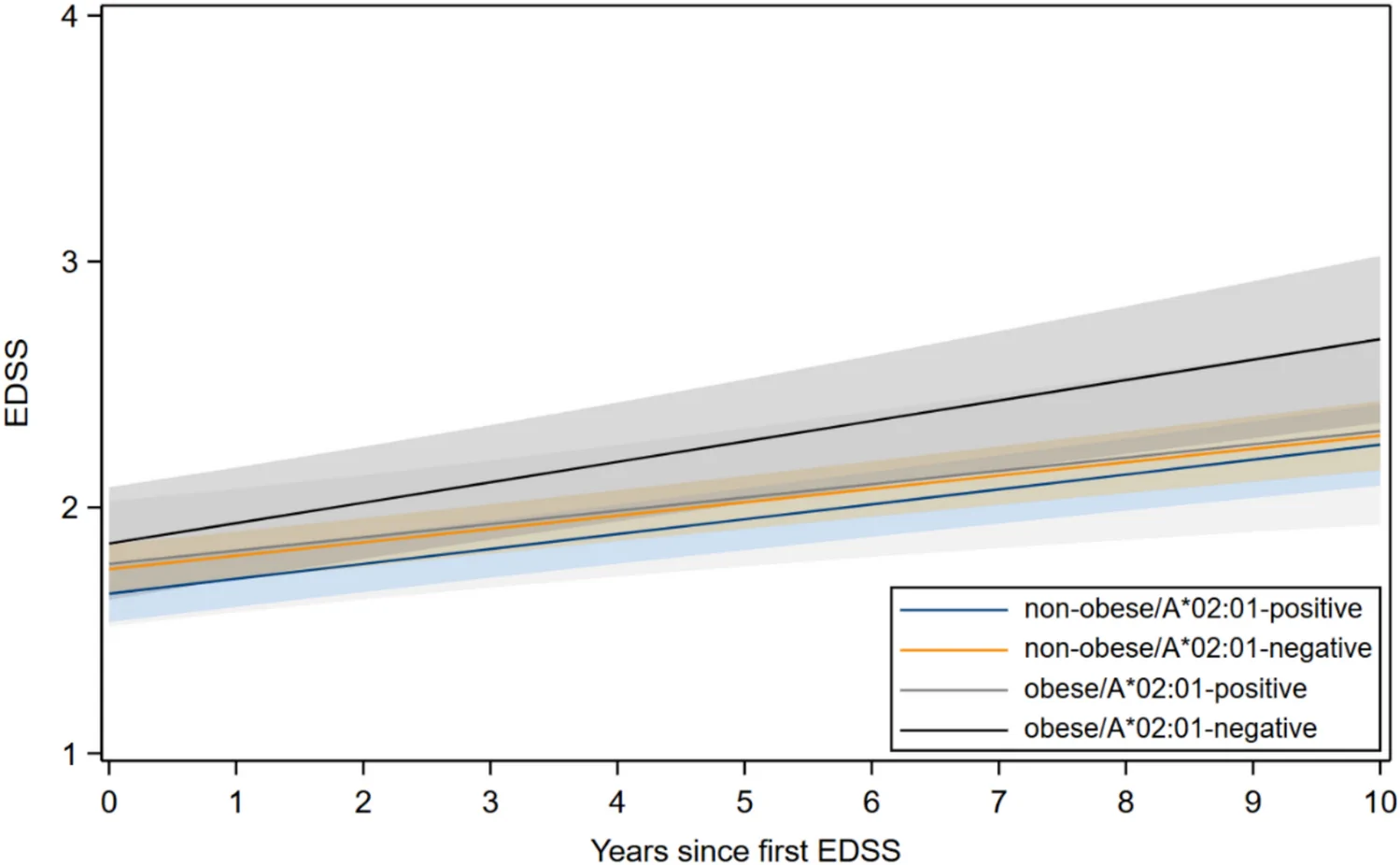
Predicted EDSS trajectories according to obesity and HLA-A*0*2:01 status. Predicted EDSS scores were derived from mixed-effects models adjusted for age at baseline, sex, ancestry, calendar year of diagnosis, disease phenotype, disease duration, and first disease-modifying therapy. Shaded areas represent 95% confidence intervals

Longitudinal mixed-effects models yielded results consistent with the time-to-event analyses. Predicted EDSS trajectories were highest among obese individuals lacking HLA-A*02:01 throughout follow-up, whereas trajectories among obese HLA-A*02:01-positive individuals were comparable to those observed in non-obese participants (Fig. 2, eTable 2). Compared with the reference group, differences in predicted EDSS among obese HLA-A*02:01-negative individuals became statistically significant from year 7 onwards (eTable 3).

Results from analyses with DMT exposure modelled as a time-varying covariate were consistent with the main findings (eTables 4). Additional adjustment for lifestyle-related covariates, including past infectious mononucleosis, alcohol use, sun exposure, and physical activity, did not meaningfully alter the observed patterns (eTables 5).

## Discussion

In this cohort of individuals with newly diagnosed MS, we observed that the association between obesity at diagnosis and subsequent disability progression differed according to HLA-A*02:01 status. Obesity was associated with a higher risk of disability worsening primarily among individuals lacking HLA-A*02:01, whereas no corresponding associations were observed among allele carriers. These findings suggest that immunogenetic background may influence the extent to which obesity contributes to long-term disability progression in MS.

Our findings extend previous observations linking obesity to adverse MS outcomes [4–6]. The present findings further suggest that the impact of obesity on disability progression is not uniform across patients, but may depend on immunogenetic background, with adverse associations observed primarily among individuals lacking HLA-A*02:01.

The relationship between obesity and disability progression is supported by several biologically plausible mechanisms. Adipose tissue is recognized as an active immuno-metabolic organ that promotes chronic low-grade inflammation, altered adipokine signaling, and immune dysregulation [20, 21]. Such alterations have been implicated across several immune-mediated inflammatory disorders, including rheumatoid arthritis and psoriatic disease [22–24]. In MS, obesity-related inflammatory pathways may contribute to sustained peripheral immune activation and promote a pro-inflammatory environment that facilitates tissue injury and disability accumulation.

HLA-A*02:01 encodes a class I major histocompatibility complex molecule involved in CD8+ T-cell antigen presentation and antiviral immune responses, particularly those directed against Epstein-Barr virus (EBV) [25, 26]. Recent evidence also suggests that HLA-A*02:01 may be associated with broader differences in immune regulation, including pathways related to type I interferon signalling [27]. The absence of HLA-A*02:01 may thus represent a broader immunogenetic context characterized by altered regulation of inflammatory pathways, thereby increasing vulnerability to obesity-related immune activation. Although the precise mechanisms require further investigation, the consistency of the observed pattern across multiple disability outcomes supports the possibility that HLA class I-mediated immune pathways contribute to the downstream effects of obesity on disease progression.

The longitudinal mixed-effects analyses provided complementary support for the time-to-event findings. Obese individuals lacking HLA-A*02:01 consistently exhibited the highest predicted EDSS scores throughout follow-up, with differences from the reference group becoming statistically significant after approximately seven years and persisting thereafter. These findings demonstrate a pattern of progressive divergence that was consistent across analytical approaches.

Obesity is increasingly recognized as a potentially important determinant of MS outcomes [4–6]. The present results support continued efforts to address obesity as part of comprehensive MS care. Beyond its potential relevance for disability progression, weight management is likely to confer substantial benefits for overall health and comorbidity prevention. Our findings further suggest that future risk stratification models may benefit from considering both lifestyle and genetic factors.

Several limitations should be acknowledged. BMI was based on self-reported height and weight and may be subject to some degree of measurement error. Such misclassification would be expected to attenuate associations rather than generate spurious interaction effects. Furthermore, BMI was only available at diagnosis, and we were, therefore, unable to evaluate how changes in body weight over time might influence disability progression. HLA were unavailable for a proportion of participants, raising the possibility of selection bias. Nonetheless, obesity prevalence at diagnosis was similar in participants with and without HLA data, indicating that missing genotypes are unlikely to have introduced major bias in the exposure-genotype analyses.

Residual confounding cannot be excluded despite adjustment for an extensive set of clinical and lifestyle factors. Finally, mechanistic interpretations remain speculative, and experimental studies will be essential to clarify how obesity-related immune pathways interact with HLA-A*02:01-dependent biology.

In summary, obesity at diagnosis showed genotype-dependent associations with disability progression, with stronger adverse effects observed among individuals lacking HLA-A*02:01. These findings extend emerging evidence that HLA class I-related immune pathways may modify the influence of environmental and lifestyle factors on MS progression and suggest that obesity may represent an important, potentially modifiable determinant of long-term disability accumulation in susceptible individuals.

## Supplementary Information

Below is the link to the electronic supplementary material.

Supplementary file1 (DOCX 680 kb)

## Data Availability

Anonymized data underlying this article will be shared upon reasonable request from any qualified investigator that wants to analyze questions that are related to the published article.

## Declarations

### Standard Protocol Approvals, Registrations, and Patient Consents

The studies were approved by the Regional Ethical Review Board at Karolinska Institute and have been performed in accordance with the ethical standards laid down in the 1964 Declaration of Helsinki and its later amendments (reference number: 04-252/1-4 and 2008/1617-31/2). Participants provided written informed consent.
