# Supplementary material for: Genetic modification of obesity-associated disability progression in multiple sclerosis
Source: J Neurol. 2026 Sep 8;273(10):578. doi: 10.1007/s00415-026-14091-7 (PMC13553611; doi:10.1007/s00415-026-14091-7)
Supplement: Supplementary file 1 [file 415_2026_14091_MOESM1_ESM.docx]

eTable 1. Measures of additive interaction (AP with 95% CI) between HLA-A*02:01 and obesity for disability outcomes.

eTable 2. Parameter estimates from mixed-effects models evaluating longitudinal EDSS trajectories according to obesity and HLA-A*02:01 status.

eTable 3. Estimated differences in mean EDSS between obese HLA-A*02:01-negative individuals and non-obese HLA-A*02:01-positive individuals derived from mixed-effects models.

eTable 4. Sensitivity analyses: Associations between obesity and disability progression outcomes post-diagnosis, by HLA-A*02:01 and obesity at diagnosis.

eTable 5. Sensitivity analysis with adjustment for lifestyle covariates: Associations between obesity and disability progression outcomes by HLA-A*02:01 obesity at diagnosis.

eFigure 1. Flow diagram of participant selection and derivation of the analytical cohort.

eTable 1. Measures of additive interaction (AP with 95% CI) between HLA-A*02:01 and obesity for disability outcomes.

| Exposure | CDW | EDSS 3 | EDSS 4 |
| --- | --- | --- | --- |
| Obesity | AP (95% CI)^1-2^ | AP (95% CI)^1-2^ | AP (95% CI)^1-2^ |
|  | **0.33 (0.03, 0.60)** | **0.46 (0.13, 0.80)** | 0.41 (-0.08, 0.89) |

HLA=human leukocyte antigen; AP=attributable proportion due to interaction; CI=confidence interval; CDW=confirmed disability worsening; EDSS=expanded disability status scale. Models were adjusted for age at baseline, sex, ancestry; calendar year of diagnosis, disease phenotype, disease duration, baseline EDSS, smoking status, and DMT exposure.

eTable 2. Parameter estimates from mixed-effects models evaluating longitudinal EDSS trajectories according to obesity and HLA-A*02:01 status.

| Obesity | HLA-A*02:01 | β (95% CI) | P value |
| --- | --- | --- | --- |
| - | + | Reference |  |
| - | - | 0.10 (-0.04,0.24) | 0.160 |
| + | + | 0.12 (-0.15,0.39) | 0.384 |
| + | - | 0.20 (-0.05,0.45) | 0.109 |
| Time | | -0.04 (-0.08,-0.01) | 0.004 |
| Time × Category | | | |
| Obesity | HLA-A*02:01 | β (95% CI) | P value |
| - | + | Reference |  |
| - | - | -0.01 (-0.02,0.01) | 0.483 |
| + | + | -0.01 (-0.04,0.03) | 0.711 |
| + | - | 0.02 (-0.01,0.05) | 0.159 |

HLA=human leukocyte antigen; CI=confidence interval. The model was adjusted for age at baseline, sex, calendar year of diagnosis, disease phenotype, disease duration, smoking status, and DMT exposure.

eTable 3. Estimated differences in mean EDSS between obese HLA-A*02:01-negative individuals and non-obese HLA-A*02:01-positive individuals derived from mixed-effects models.

| Year | Difference in mean EDSS (95% CI) | P value |
| --- | --- | --- |
| 1 | 0.05 (-0.06, 0.15) | 0.29 |
| 2 | 0.06 (-0.04, 0.16) | 0.22 |
| 3 | 0.07 (-0.03, 0.16) | 0.16 |
| 4 | 0.08 (-0.02, 0.17) | 0.11 |
| 5 | 0.09 (-0.01, 0.18) | 0.08 |
| 6 | 0.09 (-0.00, 0.19) | 0.06 |
| 7 | 0.10 (0.00, 0.20) | 0.05 |
| 8 | 0.11 (0.00, 0.21) | 0.04 |
| 9 | 0.11 (0.01, 0.22) | 0.04 |
| 10 | 0.12 (0.01, 0.24) | 0.04 |

Positive values indicate higher predicted EDSS scores among obese HLA-A*02:01-negative individuals compared with the reference group (non-obese HLA-A*02:01-positive individuals).

eTable 4. Sensitivity analyses with adjustment for DMT as a time-varying covariate: Associations between obesity and disability progression outcomes post-diagnosis, by HLA-A*02:01 and obesity at diagnosis.

| First clinical disease worsening (CDW) | | | | | | |
| --- | --- | --- | --- | --- | --- | --- |
| Obesity | A*02 | N | Years (SD) | Outcome (%) | HR (95% CI)^1^ | HR (95% CI)^2^ |
| - | + | 754 | 7.6 (6.0) | 449 (59.5) | 1.0 (reference) | 1.0 (reference) |
| - | - | 1056 | 7.8 (6.2) | 611 (57.9) | 0.97 (0.87-1.09) | 0.98 (0.91-1.06) |
| + | + | 137 | 6.8 (5.6) | 79 (57.7) | 1.93 (0.66-1.33) | 0.85 (0.63-1.03) |
| + | - | 170 | 6.2 (5.6) | 115 (67.7) | **1.37 (1.04-1.81)** | **1.44 (1.20-1.73)** |
| EDSS 3 | | | | | | |
| Obesity | A*02 | N | Years (SD) | Outcome (%) | HR (95% CI)^1^ | HR (95% CI)^2^ |
| - | + | 583 | 10.1 (6.2) | 226 (38.8) | 1.0 (reference) | 1.0 (reference) |
| - | - | 784 | 10.1 (6.3) | 315 (40.2) | 1.04 (0.89-1.23) | 0.96 (0.88-1.06) |
| + | + | 101 | 8.8 (5.8) | 46 (45.5) | 1.05 (0.63-1.74) | 0.89 (0.63-1.21) |
| + | - | 128 | 7.8 (5.7) | 70 (54.7) | **2.04 (1.40-2.98)** | **1.65 (1.30-2.08)** |
| EDSS 4 | | | | | | |
| Obesity | A*02 | N | Years (SD) | Outcome (%) | HR (95% CI)^1^ | HR (95% CI)^2^ |
| - | + | 583 | 12.1 (5.9) | 107 (18.4) | 1.0 (reference) | 1.0 (reference) |
| - | - | 784 | 12.4 (5.8) | 153 (19.5) | 1.06 (0.84-1.35) | 1.06 (0.94-1.21) |
| + | + | 101 | 11.5 (5.5) | 21 (20.8) | 1.27 (0.66-2.42) | **1.36 (1.02-2.22)** |
| + | - | 128 | 11.0 (5.5) | 36 (28.1) | **2.43 (1.46-4.05)** | **1.90 (1.40-2.57)** |

HR=hazard ratio; CI=confidence interval; CDW=confirmed disability worsening; EDSS=expanded disability status scale; HLA=human leukocyte antigen; SD=standard deviation; AP=attributable proportion due to interaction; ^1^adjusted for sex and age at baseline; ^2^adjusted for age at baseline, sex, ancestry, calendar year of diagnosis, disease phenotype, disease duration, baseline EDSS, smoking status, and DMT exposure as a time-varying covariate.

eTable 5. Sensitivity analysis with adjustment for lifestyle covariates: Associations between obesity and disability progression outcomes by HLA-A*02:01 obesity at diagnosis.

| First clinical disease worsening (CDW) | | | | | | |
| --- | --- | --- | --- | --- | --- | --- |
| Obesity | A*02 | N | Years (SD) | Outcome (%) | HR (95% CI)^1^ | HR (95% CI)^2^ |
| - | + | 754 | 7.6 (6.0) | 449 (59.5) | 1.0 (reference) | 1.0 (reference) |
| - | - | 1056 | 7.8 (6.2) | 611 (57.9) | 0.97 (0.87-1.09) | 0.98 (0.87-1.10) |
| + | + | 137 | 6.8 (5.6) | 79 (57.7) | 1.93 (0.66-1.33) | 0.90 (0.62-1.27) |
| + | - | 170 | 6.2 (5.6) | 115 (67.7) | **1.37 (1.04-1.81)** | **1.44 (1.09-1.91)** |
| EDSS 3 | | | | | | |
| Obesity | A*02 | N | Years (SD) | Outcome (%) | HR (95% CI)^1^ | HR (95% CI)^2^ |
| - | + | 583 | 10.1 (6.2) | 226 (38.8) | 1.0 (reference) | 1.0 (reference) |
| - | - | 784 | 10.1 (6.3) | 315 (40.2) | 1.04 (0.89-1.23) | 1.00 (0.85-1.18) |
| + | + | 101 | 8.8 (5.8) | 46 (45.5) | 1.05 (0.63-1.74) | 1.02 (0.62-1.70) |
| + | - | 128 | 7.8 (5.7) | 70 (54.7) | **2.04 (1.40-2.98)** | **1.82 (1.24-2.67)** |
| EDSS 4 | | | | | | |
| Obesity | A*02 | N | Years (SD) | Outcome (%) | HR (95% CI)^1^ | HR (95% CI)^2^ |
| - | + | 583 | 12.1 (5.9) | 107 (18.4) | 1.0 (reference) | 1.0 (reference) |
| - | - | 784 | 12.4 (5.8) | 153 (19.5) | 1.06 (0.84-1.35) | 1.00 (0.79-1.27) |
| + | + | 101 | 11.5 (5.5) | 21 (20.8) | 1.27 (0.66-2.42) | 1.32 (0.69-2.54) |
| + | - | 128 | 11.0 (5.5) | 36 (28.1) | **2.43 (1.46-4.05)** | **2.11 (1.26-3.54)** |

HR=hazard ratio; CI=confidence interval; CDW=confirmed disability worsening; EDSS=expanded disability status scale; HLA=human leukocyte antigen; SD=standard deviation; ^1^adjusted for sex and age at baseline; ^2^adjusted for age at baseline, sex, ancestry, calendar year of diagnosis, disease phenotype, disease duration, baseline EDSS, smoking status, past infectious mononucleosis, sun exposure, physical activity, and DMT exposure.

eFigure 1. Flow diagram of participant selection and derivation of the analytical cohort.
